# A rare clinical image of anencephaly

**DOI:** 10.11604/pamj.2023.44.41.38668

**Published:** 2023-01-23

**Authors:** Nycy Babu Sulochana, Rajkumar Gupta

**Affiliations:** 1 Department of Dravyaguna Vigyana, Mahatma Gandhi Ayurved College, Hospital and Research Centre, Salod (Hirapur), Datta Meghe Institute of Medical Sciences (Deemed to be University), Sawangi, Wardha, India

**Keywords:** Anencephaly, microcephaly, teratogenicity

## Image in medicine

Anencephaly is a medical condition where the fetus has no calvarium with no or lack of brain tissues. This belongs to neural tube defects where the neural tube fails to close. In rural areas, the lack of prenatal care and prenatal follow-up of the mother mostly leads to this malformation in the fetus. A pregnant mother of 36 weeks of gestation with no history of diabetes mellitus, epilepsy, or known exposure to teratogenic agents was taken to the hospital with labor pain. The case was taken to the C-section due to fetal distress. The baby didn’t cry immediately after the birth, resuscitation was performed immediately. There was no calvarium, no scalp, external presence of hemorrhagic tissue, and lack of cerebral hemisphere was noted. The left leg of the fetus was not developed completely. The father and family members were informed and counseled about the condition. The economic condition of the family couldn’t afford the higher medical management. The baby passed after 5 hrs of life. Counseling of the mother was done and the importance of prenatal care during pregnancy was informed to the family. Anencephaly does not support life, prevention is the only way to avoid this condition. With proper prenatal care and counseling, this can be achieved. The most important part of the treatment for the mother is counseling to overcome or avoid postpartum depression and its complications.

**Figure 1:**
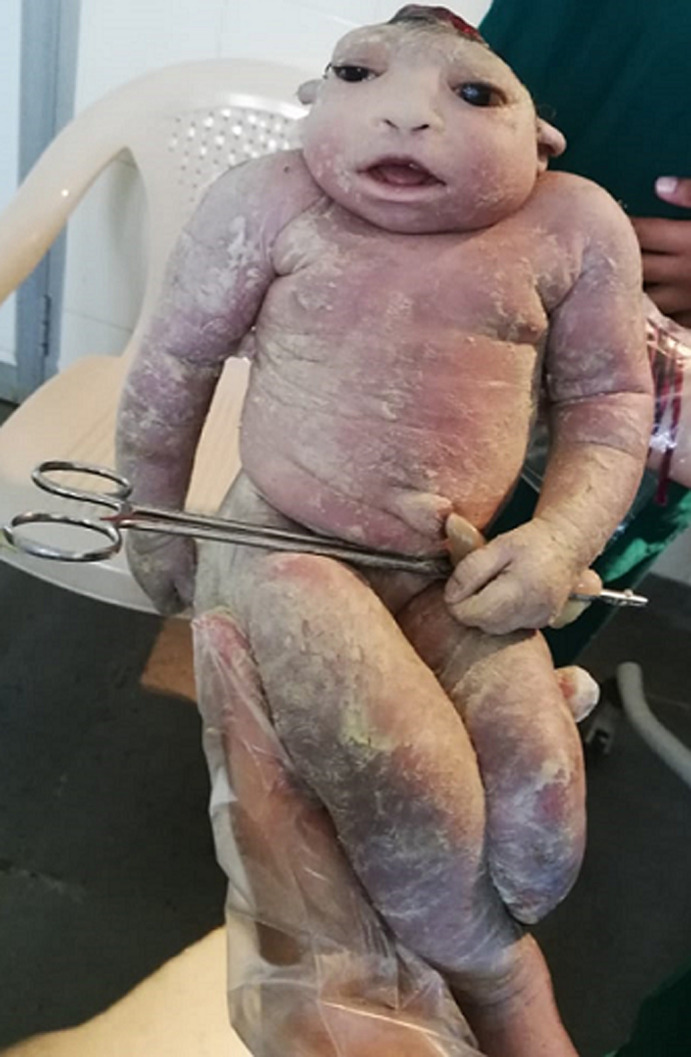
a rare clinical image of anencephaly

